# Towards Integration of Domain Knowledge-Guided Feature Engineering and Deep Feature Learning in Surface Electromyography-Based Hand Movement Recognition

**DOI:** 10.1155/2021/4454648

**Published:** 2021-12-29

**Authors:** Wentao Wei, Xuhui Hu, Hua Liu, Ming Zhou, Yan Song

**Affiliations:** ^1^School of Design Arts and Media, Nanjing University of Science and Technology, Nanjing, Jiangsu Province, China; ^2^School of Instrument Science and Engineering, Southeast University, Nanjing, Jiangsu Province, China; ^3^School of Computer Science and Engineering, Nanjing University of Science and Technology, Nanjing, Jiangsu Province, China

## Abstract

As a machine-learning-driven decision-making problem, the surface electromyography (sEMG)-based hand movement recognition is one of the key issues in robust control of noninvasive neural interfaces such as myoelectric prosthesis and rehabilitation robot. Despite the recent success in sEMG-based hand movement recognition using end-to-end deep feature learning technologies based on deep learning models, the performance of today's sEMG-based hand movement recognition system is still limited by the noisy, random, and nonstationary nature of sEMG signals and researchers have come up with a number of methods that improve sEMG-based hand movement via feature engineering. Aiming at achieving higher sEMG-based hand movement recognition accuracies while enabling a trade-off between performance and computational complexity, this study proposed a progressive fusion network (PFNet) framework, which improves sEMG-based hand movement recognition via integration of domain knowledge-guided feature engineering and deep feature learning. In particular, it learns high-level feature representations from raw sEMG signals and engineered time-frequency domain features via a feature learning network and a domain knowledge network, respectively, and then employs a 3-stage progressive fusion strategy to progressively fuse the two networks together and obtain the final decisions. Extensive experiments were conducted on five sEMG datasets to evaluate our proposed PFNet, and the experimental results showed that the proposed PFNet could achieve the average hand movement recognition accuracies of 87.8%, 85.4%, 68.3%, 71.7%, and 90.3% on the five datasets, respectively, which outperformed those achieved by the state of the arts.

## 1. Introduction

As a precise and noninvasive way of decoding user's intention of hand movements, the surface electromyography (sEMG)-based hand movement recognition has been extensively investigated in the area of rehabilitation engineering [1,2] and human-computer interaction [3,4]. Having realized that one of the key issues of sEMG-based hand movement recognition is a machine-learning-driven decision-making problem of classifying sequences of sEMG signals, many efforts have been made in improving sEMG-based hand movement recognition by designing more representative features [5], developing more sophisticated machine-learning models [6], and increasing the number of sensors [7].

From the perspective of machine learning, existing sEMG-based hand movement recognition approaches can be broadly categorized into (1) methods based on *feature engineering* and (2) methods based on *feature learning* [8]. The former refers to methods based on conventional shallow learning models and handcrafted time domain (TD), frequency domain (FD), or time-frequency domain (TFD) features, and the latter refers to methods based on end-to-end deep learning models that can learn representative high-level features from raw sEMG signals without relying on any engineered feature.

Over the past five years, feature learning approaches based on end-to-end deep learning models such as convolutional neural networks (CNNs) [9] and recurrent neural networks (RNNs) [10] have been widely studied in sEMG-based hand movement recognition. On the other hand, due to the noisy, random, and nonstationary nature of sEMG, researchers have also realized that achieving robust sEMG-based hand movement recognition accuracy remains a challenging issue for end-to-end deep learning models. For example, one of the early studies in this field revealed that the average hand movement recognition accuracy achieved by the end-to-end CNN model was significantly lower than that achieved by conventional shallow learning models such as random forests and support vector machine (SVM) on the large-scale noninvasive adaptive prosthetics (NinaPro) database [11]. Later studies on this database [12,13] presented more promising results achieved by the fine-tuned and manually optimized end-to-end deep learning models, which outperformed shallow learning models.

Compared with feature learning approaches, the hand movement recognition performance of conventional feature engineering approaches is largely dependent on the selection and extraction of features, which is usually done manually based on the domain knowledge accumulated through a vast quantity of experiments and evaluations in the field. Such heuristically accumulated domain knowledge is often thought to be useful in enhancing deep learning-based myoelectric pattern recognition [14]. Thus, a number of recent studies in this field have tried to extract and evaluate multiple engineered features as the input of their deep learning models. For example, Millar et al. [15] extracted a set of 11 TD features from sEMG signals for hand movement recognition using a long short-term memory (LSTM) model and achieved an averaged recognition accuracy of 99.8% in classifying a series of functional grasps on 2 diametric objects. Cheng et al. [16] extracted two TD features and one FD feature from sEMG signals and constructed them into the multi-sEMG feature image for hand movement recognition using a CNN model, and they achieved an averaged recognition accuracy of 82.5% in classifying 52 hand movements over 27 subjects. Allard et al. [17] evaluated different input modalities of a CNN model with transfer learning architecture and found that short-time Fourier transform-based spectrograms and continuous wavelet transform (CWT) features outperformed raw sEMG signals in classifying 7 hand movements over 17 subjects. Shen et al. [18] extracted FD and TFD features from sEMG signals, represented them by images, and used them for stacking ensemble CNN-based hand movement recognition, and they achieved an averaged recognition accuracy of 72.1% in classifying 40 hand movements over 10 subjects. Our previous study [14] extracted three sets of features from sEMG signals, constructed them into multi-view representations of sEMG signals for hand movement recognition, and achieved an averaged recognition accuracy of 83.7% in classifying 50 hand movements over 40 subjects.

To sum up, existing deep learning approaches for sEMG-based hand movement recognition can be categorized into end-to-end deep learning approaches and non-end-to-end deep learning approaches considering their input. Although the existing non-end-to-end deep learning approaches improved the sEMG-based hand movement recognition performance using engineered features instead of raw sEMG signals as their input, they to a considerable extent ignored the feature learning capability of deep learning models. In other words, their hand movement recognition performance was highly dependent on the selection of engineered features, which is usually based on domain knowledge or offline experimental results on a small set of data. Moreover, for methods that employed multiple engineered features as the input of deep learning models [14,18], the feature engineering process required additional computational time and resources, which limited their use in real-time systems.

Therefore, in this study, we propose a progressive fusion network (PFNet), which aims at improving sEMG-based hand movement recognition via progressive integration of domain knowledge-guided feature engineering and CNN-based deep feature learning. In particular, the proposed PFNet architecture is composed of three parts, namely the feature learning network, the domain knowledge network, and the progressive fusion module. The feature learning network and the domain knowledge network learn high-level feature representations from raw sEMG signals and engineered features, respectively, and the two networks are progressively integrated together via a 3-stage process in the progressive fusion module.

The major contributions of the proposed PFNet architecture are twofold:

1. We built up two independent neural networks, namely the feature learning network and the domain knowledge network, to separately learn discriminative high-level feature representations from raw sEMG signals and the wavelet packet-based TFD features that have been proven to be effective for sEMG-based hand movement recognition in early studies; thus, the hand movement recognition performance can be improved with the help of both deep feature learning and heuristically accumulated domain knowledge.
2. We employed a 3-stage process to progressive integrated domain knowledge-guided feature engineering and deep feature learning in sEMG-based hand movement recognition. In particular, feature-level fusion was performed at first to fuse the high-level feature representations learned at two different depths of the feature learning network and the domain knowledge network together via two subnetworks, and then, the output decisions of the two subnetworks were fused together through decision-level fusion. Such a 3-stage integration strategy is believed to be capable of learning more diverse high-level feature representations, which is helpful for improving the hand movement recognition performance.

The experimental results on five datasets not only proved the effectiveness of integration of domain knowledge-guided feature engineering and deep feature learning in sEMG-based hand movement recognition, but also indicated that our approach outperformed other state-of-the-art methods.

## 2. Materials and Methods

### 2.1. Datasets and Data Preprocessing

Experiments in this study were carried out on 5 subdatasets of the NinaPro repository [19], which provides publicly available multichannel sEMG signals recorded from intact subjects and trans-radial amputees.  Table 1 presents brief information of the sEMG datasets used in this study, and detailed descriptions are as follows:

The first subdataset of NinaPro (denoted as NinaProDB1) provides 10-channel sEMG signals collected from 53 hand movements performed by 27 healthy subjects. The hand movements in NinaProDB1 were categorized into 12 finger movements (denoted as Exercise A), 17 wrist movements and hand postures (denoted as Exercise B), 23 grasping and functional movements (denoted as Exercise C), and the rest movement, and each hand movement was repeated 10 times (i.e., 10 trials per hand movement) [20]. As most of the existing studies on this NinaProDB1 excluded the rest movement from their experiments [10,12,14,22], in our experiments we also excluded the rest movement for the convenience of performance comparison.

The second subdataset of NinaPro (denoted as NinaProDB2) provides 12-channel sEMG signals collected from 50 hand movements performed by 40 healthy subjects. The hand movements in NinaProDB2 were categorized into 17 wrist movements and hand postures (i.e., as same as Exercise B in NinaProDB1), 23 grasping and functional movements (i.e., as same as Exercise C in NinaProDB1), 9 force patterns (denoted as Exercise D), and the rest movement, and each hand movement was repeated 6 times (i.e., 6 trials per hand movement) [20].

The third subdataset of NinaPro (denoted as NinaProDB3) provides 12-channel sEMG signals collected from 50 hand movements performed by 11 trans-radial amputee subjects. The hand movements in NinaProDB3 are exactly the same as those in NinaProDB2, and each hand movement was repeated 6 times (i.e., 6 trials per hand movement) [20]. According to Atzori et al. [20], during the data recording process of NinaProDB3 three trans-radial amputee subjects interrupted the experiment before its end due to fatigue or pain, and two trans-radial amputee subjects used only 10 electrodes to collect sEMG signals due to insufficient space. The data from these subjects were omitted in our experiments to ensure that the number of hand movement repetitions, as well as the number of sEMG channels for each subject, was the same.

The fourth subdataset of NinaPro (denoted as NinaProDB4) provides 12-channel sEMG signals collected from 53 hand movements performed by 10 healthy subjects. The hand movements in NinaProDB4 are exactly the same as those in NinaProDB1, and each hand movement was repeated 6 times (i.e., 6 trials per hand movement) [21]. After checking the data, we found that two subjects (i.e., subject 4 and subject 6) did not complete all hand movements, and their data were omitted in our experiments.

The fifth subdataset of NinaPro (denoted as NinaProDB5) provides 16-channel sEMG signals collected from 53 hand movements performed by 10 healthy subjects. The hand movements in NinaProDB5 are exactly the same as those in NinaProDB1, and each hand movement was repeated 6 times (i.e., 6 trials per hand movement) [21]. A subset of 41 hand movements were classified in our experiments, and the specifications of the selected hand movements can be found in [21].

The sEMG signals in NinaProDB1 were recorded by Otto Bock 13E200-50 electrodes at a sampling rate of 100 Hz, the sEMG signals in NinaProDB2 and DB3 were recorded by Delsys Trigno Wireless electrodes at a sampling rate of 2k Hz, and the sEMG signals in NinaProDB4 were recorded by Cometa Wave Plus Wireless sEMG system at a sampling rate of 2k Hz [20,21]. Because of the memory limitation, we downsampled the sEMG signals in NinaProDB2-NinaProDB4 from 2k Hz to 100 Hz. The same experimental configuration was also adopted in [14].

The raw sEMG signals in each dataset were segmented by sliding windows. As early studies [23,24] have indicated that the maximum allowable time delay of real-time myoelectric control systems is 300 ms, and for all experiments in this study, we employed sliding windows that were no longer than 200 ms to segment raw sEMG signals. Detailed information of the sliding window lengths and steps used in this study will be presented in the results and discussion section of this study.

### 2.2. Domain Knowledge-Guided Feature Engineering and Feature Augmentation

**Figure 1:**
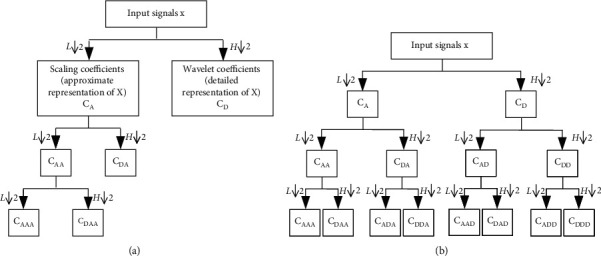
A schematic diagram of (a) DWT and (b) DWPT with the wavelet level of 3, where *L*↓2 denotes the half-band low-pass filter and *H*↓2 denotes the half-band high-pass filter. *C*_AA_ denotes the approximate representation (scaling coefficients) of *C*_*A*_, *C*_DA_ denotes the detailed representation (wavelet coefficients) of *C*_*A*_, and so on.

Discrete wavelet transform (DWT) is a time-frequency analysis approach that iteratively decomposes the original discrete time series into wavelet coefficients in multiresolution sub-bands via a set of half-band filters that are established based on a pair of orthogonal wavelet basis functions [25]. As shown in Figure 1(a), at the first wavelet level, a half-band low-pass filter and a half-band high-pass filter decompose the original signals *X* into two sequences of coefficients in the lower resolution space, namely the scaling coefficients *C*_*A*_, which are the approximate representation of *X*, and wavelet coefficients *C*_*D*_, which are the detailed representation of *X*, respectively. Such process is iteratively repeated on the decomposed scaling coefficients at the subsequent wavelet levels, resulting in a two-channel tree structure that subsamples the signals by 2 at each node.

The discrete wavelet packet transform (DWPT) is an extension of DWT, in which not only scaling coefficients but also wavelet coefficients are decomposed into two sequences of coefficients in the lower resolution space at each wavelet level. As shown in Figure 1(b), when the wavelet level *k* = 3, the outputs of DWPT are composed of a total of 2^3^ = 8 sequences of DWPT coefficients (DWPTCs), which can be regarded as the multiresolution representation of original signals *X* in 8 sub-bands.

The DWPT has been widely used in sEMG-based hand movement recognition as a feature engineering technique for the extraction of TFD features. Conventional shallow learning methods usually extract statistic features, such as energy, average value, standard deviation, skewness, and kurtosis. Conventional shallow learning methods usually extract statistic features, such as energy, average value, standard deviation, skewness, and kurtosis from DWPTCs as the input of their classifiers [26, 27], while most of the state of the arts adopt the strategy of using images generated from DWPTCs in all sub-bands to form the input of deep neural networks [14, 18]. In our previous study [14], a total of 11 engineered features and feature sets were evaluated as the input of a CNN model for sEMG-based hand movement recognition, and the results showed that the hand movement recognition accuracy achieved by DWPTCs on different datasets outperformed all other features and feature sets.

Based on the aforementioned domain knowledge, the DWPTCs were extracted from raw sEMG signals in this study to generate the input images of the domain knowledge network. The DWPT hyperparameters used in this study are exactly the same as those used in our previous study [14]. In particular, we used the Daubechies 1 wavelet basis function, and the wavelet level *k* was set to⌊log  2^*N*^⌋, where *N* is the length of input signals (i.e., length of the sliding window). For each sEMG channel, the resulting 2^*k*^ DWPTC sequences in all sub-bands were concatenated together to form a DWPTC vector, and the DWPTC vectors from all sEMG channels were stacked into a DWPTC image.

Two DWPTC images extracted from each sliding window were further augmented by the algorithm proposed by Jiang and Yin [28]. Such feature augmentation strategy, which was also adopted in our previous study [14], enables every sEMG channel to have a chance to be adjacent to every other channel via channel reorganization, thus providing additional spatial correlations between nonadjacent sEMG channels for the deep learning model. Suppose the DWPTC image extracted from each frame sliding window has a shape of *D* × *C*, where *C* is the number of sEMG channels, the *D* × *C* DWPTC image was reorganized into an *D* × *M* image after feature augmentation. When *C* = 10, we have *M* = 50, and when *C* = 12, we have *M* = 72.

### 2.3. Proposed PFNet Architecture

**Figure 2:**
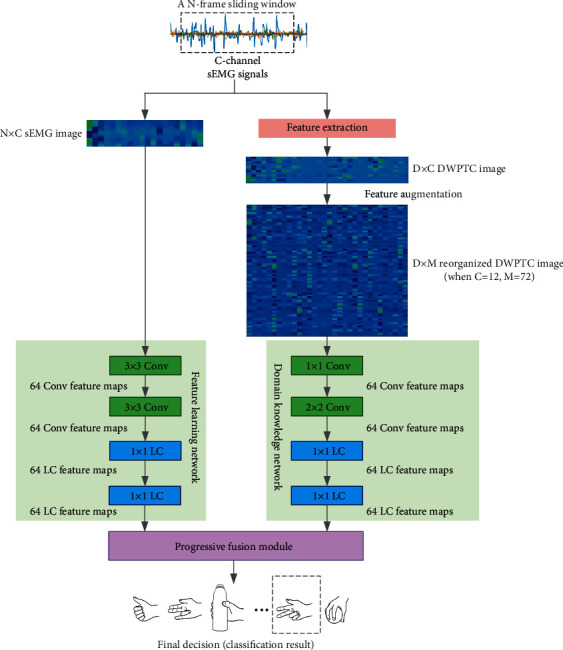
A schematic drawing demonstrating our proposed PFNet. The boxes marked with “Conv” and “LC” denote the convolutional layers and the locally connected layers, respectively.

Figure 2 demonstrates the architecture of our proposed PFNet, which consists of a feature learning network, a domain knowledge network, and the progressive fusion module. Suppose *N*-frame sliding windows are used to segment *C*-channel sEMG signals, the input images of feature learning network are N × C sEMG images, which are formed by stacking C-channel raw sEMG signals together, and the input images of the domain knowledge network are D × M reorganized DWPTC images, which has been discussed in the previous subsection.

### 2.4. Feature Learning Network

The feature learning network performs feature learning on raw sEMG signals, and it is composed of two convolutional layers with 3 × 3 filters, two locally connected layers with 11 filters, and one fully connected layer with 512 hidden units. The number of output feature maps of every neural network layer in the feature learning network was set to 64. The feature learning network shares the same architecture with the first four neural network layers of GengNet [12], which showed promising sEMG-based hand movement recognition performance in existing studies [12–14].

### 2.5. Domain Knowledge Network

The domain knowledge network learns high-level feature representations from reorganized DWPTC images. The network architecture of domain knowledge network is slightly different from the feature learning network, and it is composed of one convolutional layer with 1 × 1 filters, one convolutional layer with 2 × 2 filters, two locally connected layers with 1 × 1 filters, and one fully connected layer with 1024 hidden units. The number of output feature maps of every neural network layer in the domain knowledge network was also set to 64.

### 2.6. Progressive Fusion Module

Conventional fusion methods for dealing with feature vectors obtained from multiple sources can be categorized into feature-level fusion and decision-level fusion, and the former concatenates the feature vectors and feeds the resulting feature vector into the classifier, while the latter builds up independent classifiers for feature vector from each data source and then fuses the decisions together to form the final decisions [29].

**Figure 3:**
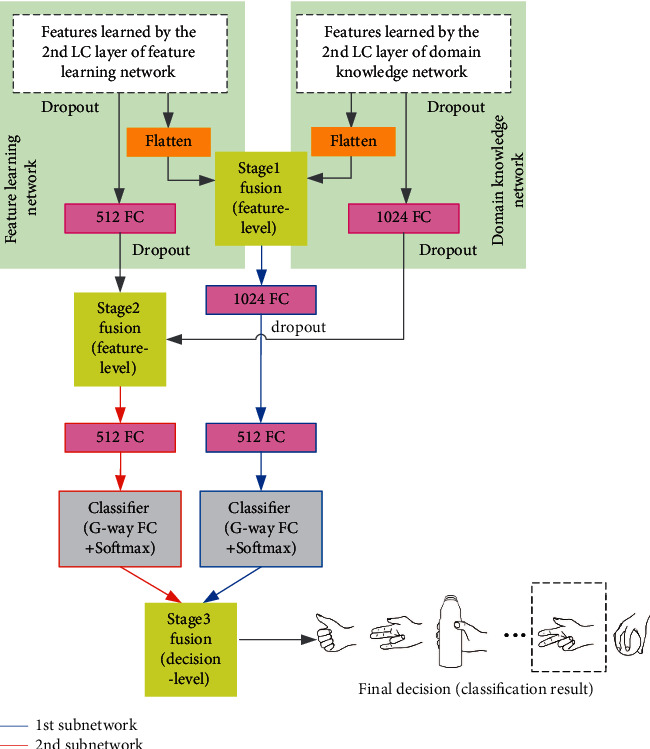
A schematic drawing demonstrating the progressive fusion module in our proposed PFNet. The boxes marked with “FC” denote the fully connected layers, and the numbers before “FC” denote their number of hidden units. The 3-stage progressive fusion process is highlighted by yellow boxes, and the 1st and 2nd subnetworks for feature-level fusion are marked with blue and red lines, respectively.

In this study, we proposed the progressive fusion module as shown in Figure 3 for fusion of feature learning network and domain knowledge network, which is able to obtain more diverse high-level feature representations via a 3-stage fusion process. Suppose F_4_^*f*^ and F_4_^*d*^ denote the flattened feature maps learned by the 4th neural network layers (i.e., the 2nd locally connected layers) of feature learning network and domain knowledge network, respectively, and F_5_^*f*^ and F_5_^*d*^ denote the feature vectors learned by the 5th neural network layers (i.e., the 1st fully connected layers) of feature learning network and domain knowledge network, respectively, the 3-stage fusion process can be formulated as follows.

1st-stage fusion (feature-level fusion):
  (1) $$\begin{array}{c} y_{1}=H_{1}\left(\left.F_{4}^{f}\right\|F_{4}^{d};\theta_{1}\right). \end{array}$$
2nd-stage fusion (feature-level fusion):
  (2) $$\begin{array}{c} y_{2}=H_{2}\left(\left.F_{5}^{f}\right\|F_{5}^{d};\theta_{2}\right). \end{array}$$
3rd-stage fusion (decision-level fusion):
  (3) $$\begin{array}{c} y_{\text{final}}=y_{1}⊕y_{2}. \end{array}$$

Here, || denotes the concatenation operation and ⊕ denotes the element-wise summation operation, H_*i*_(*i*=1,2) are two subnetworks for feature-level fusion of high-level features learned at two different depths of feature learning network and domain knowledge network, and *θ*_*i*_ and *y*_*i*_ refer to their parameters and output decisions, respectively. As shown in equation (3), the output decisions of two subnetworks H_1_ and H_2_, which are in the form of softmax scores, are finally summed up at the 3rd-stage fusion to obtain the final decision (classification result).

For a more distinct view of the two subnetworks for feature-level fusion in the 3-stage progressive fusion process, we marked the 1st and 2nd subnetworks (i.e., H_1_ and H_2_) with blue and red lines, respectively, in Figure 3.

### 2.7. Neural Network Configurations and Hyperparameter Settings

We applied batch normalization [30] to each neural network layer of the PFNet to reduce the internal covariate shift and rectified linear unit (ReLU) activation function [31] after each neural network layer to fasten the training process. As shown in Figure 3, we also applied dropout regularization [32] after five neural network layers (i.e., the 2nd locally connected layers and the 1st fully connected layers of the feature learning network and the domain knowledge network, as well as the 1st fully connected layer of the 1st subnetwork H_1_) to avoid overfitting.

To prevent overfitting, for all experiments in this study we employed a pre-training strategy that has been widely used in sEMG-based hand movement recognition systems [10,12–14,33]. In particular, during each experiment, we firstly pre-trained a model using all available training data and then used the pre-trained model as the initial model in each fold of the validation. The pre-training and training were based on stochastic gradient descent (SGD) algorithm with batch size of 1000, and the number of training epochs was set to 28. To improve convergence, we also applied a learning rate decay strategy [34], which initialized the learning rate at 0.1 and divided it by 10 at the 16th and 24th epochs, respectively. For layers with dropout regularization, the dropout rate was set to 0.5 during pre-training and set to 0.65 during training.

### 2.8. Evaluation Metrics

For the convenience of performance comparison, the evaluation metrics used in this study were the same as those used in existing studies on the NinaPro dataset [10,12,14,20,22,33,35]. In particular, we followed the intra-subject classification schemes proposed by the author of NinaPro dataset [20,21], which used sEMG signals from approximately 2/3 of the hand movement repetitions performed by each subject as the training set and sEMG signals from the remaining hand movement repetitions performed by the same subject as the test set. The final hand movement recognition accuracy on each dataset is obtained by averaging the achieved accuracies over all subjects.

The selection of training and test set on different subdatasets of NinaPro can be described as follows:

  NinaProDB1: the sEMG signals from the 1st, 3rd, 4th, 6th, 7th, 8th, and 9th repetitions of all hand movements are used as the training set, while the sEMG signals from the 2nd, 5th, and 10th repetitions of all hand movements constitute the test set.
  NinaProDB2, NinaProDB3, NinaProDB4, and NinaProDB5: the sEMG signals from the 1st, 3rd, 4th, and 6th repetitions of all hand movements are used as the training set, while the sEMG signals from the 2nd and 5th repetitions of all hand movements constitute the test set.

## 3. Results and Discussion

### 3.1. Computational Time and Efficiency

All experiments in this study were performed offline with MXNet [36] on a NVIDIA GeForce GTX 1080 Ti GPU. In our experiments, the hardware factors that affected the computational time and training speed include not only GPU utilization percentage, but also the network throughput, as all of the offline experimental data (i.e., sEMG signals) are stored on a network-attached storage (NAS) device; thus, it is hard to estimate the computational time of our proposed PFNet for sEMG-based hand movement recognition in real-world scenarios. Even so, we calculated the approximate computational time and efficiency for training, which are as follows.

The training of each fold (i.e., each subject) of intra-subject evaluation took approximately 23–30 minutes on NinaProDB1, 11–17 minutes on NinaProDB2, 18–20 minutes on NinaProDB3, 37–39 minutes on NinaProDB4, and 3–4 minutes on NinaProDB5, and the training speed on NinaProDB1, NinaProDB2, NinaProDB3, NinaProDB4, and NinaProDB5 was approximately 3500 samples per second, 3300 samples per second, 6400 samples per second, 3300 samples per second, and 3500 samples per second, respectively.

### 3.2. Ablation Studies on the Proposed Method

**Figure 4:**
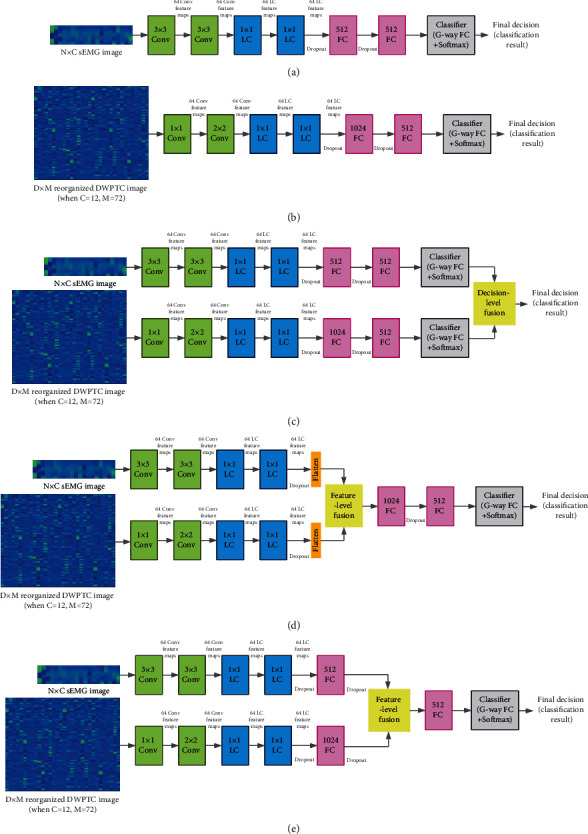
A schematic drawing demonstrating the deep neural network architectures evaluated in the ablation studies, including (a) the FLonly architecture, (b) the DKonly architecture for Ablation Study 1, and neural network architectures of (c) decision-level fusion, (d) stage1 feature-level fusion, and (e) stage2 feature-level fusion for Ablation Study 2.

In machine learning, “ablation studies” usually refer to a procedure to evaluate certain parts of the deep neural network, where the other parts of the deep neural network are removed from the evaluation. In this study, we conducted two ablation studies on the proposed PFNet to verify its effectiveness, which can be described as follows:

1. Ablation Study 1: a performance comparison among the proposed PFNet, PFNet without the domain knowledge network and its input (denoted as FLonly), and PFNet without the feature learning network and its input (denoted as DKonly), to verify the effectiveness of integration of domain knowledge-guided feature engineering and deep feature learning in sEMG-based hand movement recognition. The neural network architectures of FLonly and DKonly are illustrated in Figures 4(a) and 4(b), respectively.
2. Ablation Study 2: a performance comparison among different approaches for fusion of feature learning network and domain knowledge network, including the proposed progressive fusion module, the decision-level (i.e., score) fusion approach, and two feature-level fusion approaches.

For all experiments in these ablation studies, the sliding window length was set to 200 ms, and the window step was set to 10 ms except for experiments on NinaProDB5, in which we followed the experimental configuration used by Pizzolato et al. [21] and our previous study [14] that set the window step to 100 ms.

**Figure 5:**
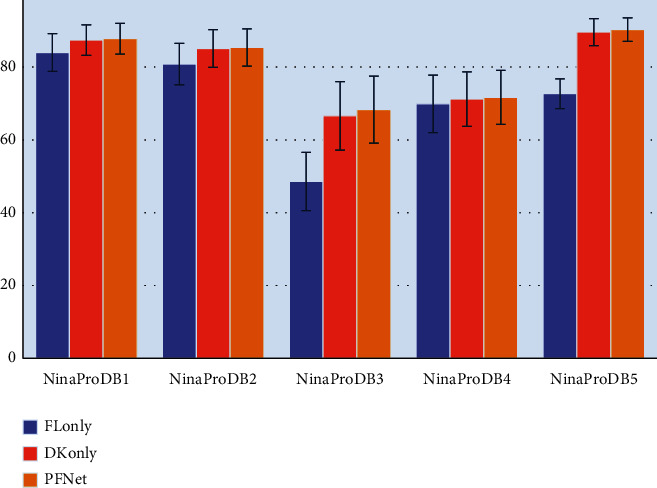
Average hand movement recognition accuracies achieved by the FLonly architecture, the DKonly architecture, and our proposed PFNet on NinaProDB1–NinaProDB5, when the sliding window length was set to 200 ms.

Figure 5 demonstrates the average hand movement recognition accuracies achieved by FLonly, DKonly, and our proposed PFNet. The experimental results showed that our proposed PFNet outperformed both FLonly and DKonly on all datasets (i.e., NinaProDB1-NinaProDB5). In particular, the average hand movement recognition accuracies achieved by our proposed PFNet were 87.8 ± 4.2%, 85.4 ± 5.1%, 68.3 ± 9.2%, 71.7 ± 7.4%, and 90.3 ± 3.2% on NinaProDB1, NinaProDB2, NinaProDB3, NinaProDB4, and NinaProDB5, respectively, which were much higher than those achieved by the FLonly architecture (i.e., 84.0 ± 5.2%, 80.8 ± 5.7%, 48.6 ± 8.0%, 69.9 ± 7.9%, and 72.7 ± 4.1% on NinaProDB1, NinaProDB2, NinaProDB3, NinaProDB4, and NinaProDB5, respectively). Compared with FLonly, the average hand movement recognition accuracies achieved by the DKonly architecture were much closer to, but also significantly outperformed by those achieved by the proposed PFNet, which were 87.4 ± 4.2%, 85.1 ± 5.2%, 66.6 ± 9.4%, 71.2 ± 7.5%, and 89.6 ± 3.7% on NinaProDB1, NinaProDB2, NinaProDB3, NinaProDB4, and NinaProDB5, respectively.

The experimental results in Ablation Study 1 showed that the integration of domain knowledge-guided feature engineering and deep feature learning is an effective way of improving sEMG-based hand movement recognition. Although the increase in input data may increase computational complexity, the computational time and training speed presented in Section 3.1 are still acceptable for real-world sEMG-based hand movement recognition systems. Moreover, compared with other deep learning methods that relied only on domain knowledge-guided feature engineering [14, 18], the integration of domain knowledge-guided feature engineering and deep feature learning achieves the balance between hand movement recognition performance and computational complexity, which is meaningful for real-time application scenarios.

In Ablation Study 2, we carried out a performance comparison among different methods for fusion of feature learning network and domain knowledge network, including our proposed progressive fusion module, the decision-level (i.e., score) fusion approach (as illustrated in Figure 4(c)), a feature-level fusion approach (denoted as stage 1 feature-level fusion, as illustrated in Figure 4(d)) that is equivalent to PFNet without stage 2 fusion and stage 3 fusion, and a feature-level fusion approach (denoted as stage 2 feature-level fusion, as illustrated in Figure 4(e)) that is equivalent to PFNet without stage 1 fusion and stage 3 fusion. For the decision-level fusion approach, the number of hidden units of 2nd fully connected layer in both feature learning network and domain knowledge network was set to 512, which is exactly the same as the number of hidden units of the second last fully connected layers in the 1st and 2nd subnetworks.

**Figure 6:**
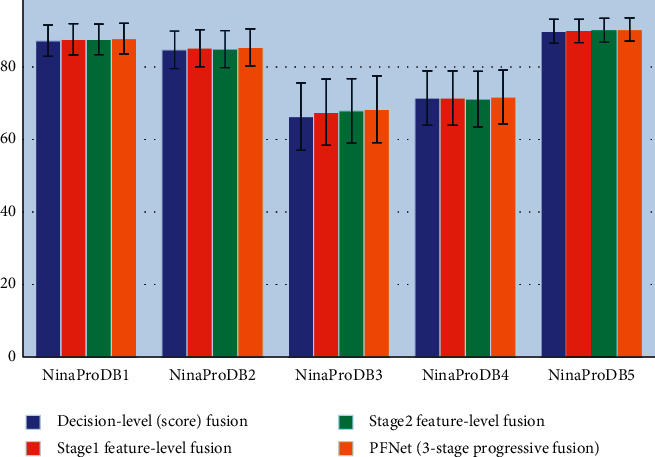
Average hand movement recognition accuracies achieved by decision-level fusion, stage 1 feature-level fusion, stage 2 feature-level fusion, and our proposed PFNet on NinaProDB1–NinaProDB5, when the sliding window length was set to 200 ms.

Figure 6 demonstrates the average hand movement recognition accuracies achieved by decision-level fusion, stage 1 feature-level fusion, stage 2 feature-level fusion, and our proposed PFNet. According to the experimental results, the 3-stage progressive fusion was able to achieve higher sEMG-based hand movement recognition accuracies than the conventional single-stage feature-level fusion (e.g., stage 1 feature-level fusion and stage 2 feature-level fusion) approaches or decision-level fusion approach. However, we also found that the performance gap between the proposed progressive fusion module and conventional fusion approaches was not significant. For example, stage 1 feature-level fusion achieved the hand movement recognition accuracies of 87.6 ± 4.3%, 85.2 ± 5.1%, 67.5 ± 9.1%, 71.4 ± 7.5%, and 89.9 ± 3.2% on NinaProDB1, NinaProDB2, NinaProDB3, NinaProDB4, and NinaProDB5, respectively, which were very close to those achieved by the PFNet. The subtle performance gap between different fusion methods indicates that the convolutional and locally connected layers at the bottom of feature learning network and domain knowledge network may play a more dominant role in sEMG-based hand movement recognition.

### 3.3. Comparison with the State of the Arts

We also compared the average hand movement recognition accuracies achieved by the proposed PFNet with those achieved by the state of the arts. For a fair performance comparison, we only considered the state of the arts that used the same intra-subject classification schemes as described in Section 2.4, and we evaluated the hand movement recognition accuracies achieved with sliding windows of 50 ms, 100 ms, 150 ms, and 200 ms. Window step settings were the same as those used in the ablation studies, except for experiments on NinaProDB5 with 50 ms, 100 ms, and 150 ms sliding windows, in which we set the window step to 10 ms.

Table 2 presents the hand movement recognition accuracies achieved by our proposed PFNet and the state of the arts on NinaProDB1, NinaProDB2, NinaProDB3, NinaProDB4, and NinaProDB5. According to the experimental results, our proposed PFNet achieved higher hand movement recognition accuracies than all the state-of-the-art deep learning methods [10–14, 16, 18, 22, 37, 38] and shallow learning methods [20, 21] listed in Table 2 on NinaProDB2, NinaProDB3, NinaProDB4, and NinaProDB5. On NinaProDB1, our proposed PFNet was outperformed by MV-CNN, which was proposed in our previous study [14]. On the other hand, it should be mentioned that MV-CNN is a multi-view deep learning method that used three high-dimensional feature sets as its input, and the performance gap between PFNet and MV-CNN was insignificant on NinaProDB1. These results indicate that our proposed PFNet framework can effectively improve sEMG-based hand movement recognition with the help of both feature learning and domain knowledge-guided feature engineering.

## 4. Conclusion

Aiming at improving sEMG-based hand movement recognition, this study proposed a progressive fusion network (PFNet) framework, which learns high-level feature representations from raw sEMG signals and discrete wavelet packet transform coefficients (DWPTCs) via a feature learning network and a domain knowledge network, respectively, and then employs a progressive fusion module to fuse the two networks together via a 3-stage process and obtain the final decisions.

Ablation studies were conducted on five open-source sEMG datasets (i.e., NinaProDB1-NinaProDB5), and the experimental results proved the effectiveness of integration of domain knowledge-guided feature engineering and deep feature learning in sEMG-based hand movement recognition, as well as the effectiveness of the proposed progressive fusion module.

Moreover, we also carried out performance comparison with the state of the arts on NinaProDB1-NinaProDB5. The experimental results showed that the proposed PFNet could achieve the average hand movement recognition accuracies of 87.8 ± 4.2%, 85.4 ± 5..1%, 68.3 ± 9.2%, 71.7 ± 7.4%, and 90.3 ± 3.2% on NinaProDB1, NinaProDB2, NinaProDB3, NinaProDB4, and NinaProDB5, respectively, which outperformed those achieved by the state-of-the-art methods on most of the evaluated datasets. Compared with our recently proposed method that used multiple engineered feature sets as its input [14], our proposed PFNet could achieve higher or almost the same hand movement recognition accuracies with only one type of engineered feature.

Future improvement of the proposed PFNet framework will focus on simplification of the deep neural network architecture while maintaining its performance, as real-time sEMG-based hand movement recognition systems usually required a more lightweight machine-learning model with fewer parameters and less computational complexity.

## Figures and Tables

**Table 1:** Brief information of the sEMG datasets used in this study.

Datasets	Num. of hand movements	Num. of subjects	Num. of sEMG channels	Type of electrodes	Num. of movement repetitions
NinaProDB1 [20]	53	27 (healthy)	10	Otto Bock	10
NinaProDB2 [20]	50	40 (healthy)	12	Delsys Trigno	6
NinaProDB3 [20]	50	11 (amputee)	12	Delsys Trigno	6
NinaProDB4 [21]	53	10 (healthy)	12	Cometa Wave	6
NinaProDB5 [21]	41	10 (healthy)	16	Thalmic MYO	6

“Num.” is short for number.

**Table 2:** Average hand movement recognition accuracies in comparison with the state of the arts on NinaProDB1-NinaProDB5.

Dataset	Machine-learning (ML) model	Type of ML model	Input of ML model	Num. of movements for classification	Window length			
					50 ms	100 ms	150 ms	200 ms
NinaProDB1	Random forests [20]	Shallow learning	Incell 5 engineered features	50	N.A.	N.A.	N.A.	75.3%
	GengNet [12]	CNN	Raw sEMG	52	N.A.	N.A.	N.A.	77.8%
	AtzoriNet [11]	CNN	Raw sEMG	50	N.A.	N.A.	66.6% ± 6.4%	N.A.
	WeiNet [13]	CNN	Raw sEMG	52	81.7%	83.4%	84.4%	85.0%
	HuNet [10]	CNN-RNN	Phinyomark feature set	52	N.A.	N.A.	86.8%	87.0%
	MV-CNN [14]	CNN	3 feature sets	52	85.8%	86.8%	87.4%	88.2%
	Evolved CNN [22]	CNN	Raw sEMG	52	N.A.	N.A.	N.A.	81.4%
	ChengNet [16]	CNN	Multi-sEMG feature image	52	N.A.	N.A.	N.A.	82.5%
	**PFNet**	**CNN**	**Raw sEMG** **+** **DWPTC**	**52**	**85.1** ± 4.6%	**86.1** ± 4.4%	**87.0** ± 4.3%	**87.8** ± 4.2%

NinaProDB2	Random forests [20]	Shallow learning	5 engineered features	50	N.A.	N.A.	N.A.	75.3%
	AtzoriNet [11]	CNN	Raw sEMG	50	N.A.	N.A.	60.3 ± 7.7%	N.A.
	ZhaiNet [37]	CNN	sEMG spectrogram	50	N.A.	N.A.	N.A.	78.7%
	HuNet [10]	CNN-RNN	Phinyomark feature set	50	N.A.	N.A.	N.A.	82.2%
	MV-CNN [14]	CNN	3 feature sets	50	80.6%	81.1%	82.7%	83.7%
	Evolved CNN [22]	CNN	Raw sEMG	50	N.A.	71.0%	N.A.	71.6%
	**PFNet**	**CNN**	**Raw sEMG** **+** **DWPTC**	**50**	**82.4** ± 5.6%	**83.4** ± 5.5%	**84.4** ± 5.6%	**85.4** ± 5.1%

NinaProDB3	SVM [20]	Shallow learning	5 handcrafted features	50	N.A.	N.A.	N.A.	46.3%
	MV-CNN [14]	CNN	3 feature sets	50	N.A.	N.A.	N.A.	64.3%
	**PFNet**	**CNN**	**Raw sEMG** **+** **DWPTC**	**50**	**64.8** ± 8.9%	**66.3** ± 9.0%	**67.3** ± 8.9%	**68.3** ± 9.2%

NinaProDB4	Random forests [21]	Shallow learning	mDWT features	53	N.A.	N.A.	N.A.	69.1%
	MV-CNN [14]	CNN	3 feature sets	53	N.A.	N.A.	N.A.	54.3%
	**PFNet**	**CNN**	**Raw sEMG** **+** **DWPTC**	**53**	**60.0** ± 8.2%	**65.8** ± 7.7%	**69.1** ± 7.5%	**71.7** ± 7.4%

NinaProDB5	SVM [21]	Shallow learning	mDWT features	41	N.A.	N.A.	N.A.	69.0%
	ShenNet [18]	Stacking-based CNN	TD, FD, and TFD feature images	40	N.A.	N.A.	N.A.	72.1%
	MV-CNN [14]	CNN	3 feature sets	41	N.A.	N.A.	N.A.	90.0%
	**PFNet**	**CNN**	**Raw sEMG** **+** **DWPTC**	**41**	**89.1** ± 3.6%	**89.6** ± 3.4%	**90.2** ± 3.3%	**90.3** ± 3.2%

N.A. denotes not applicable, and bold entries indicate our proposed method.

## Data Availability

The sEMG signals supporting the findings of this study are from the NinaPro dataset, which is publicly available at ninapro.hevs.ch. The papers describing the NinaPro dataset are cited at relevant places within the text as references [20, 21]. The processed data and trained deep neural networks used to support the findings of this study are available from the corresponding author upon request.
